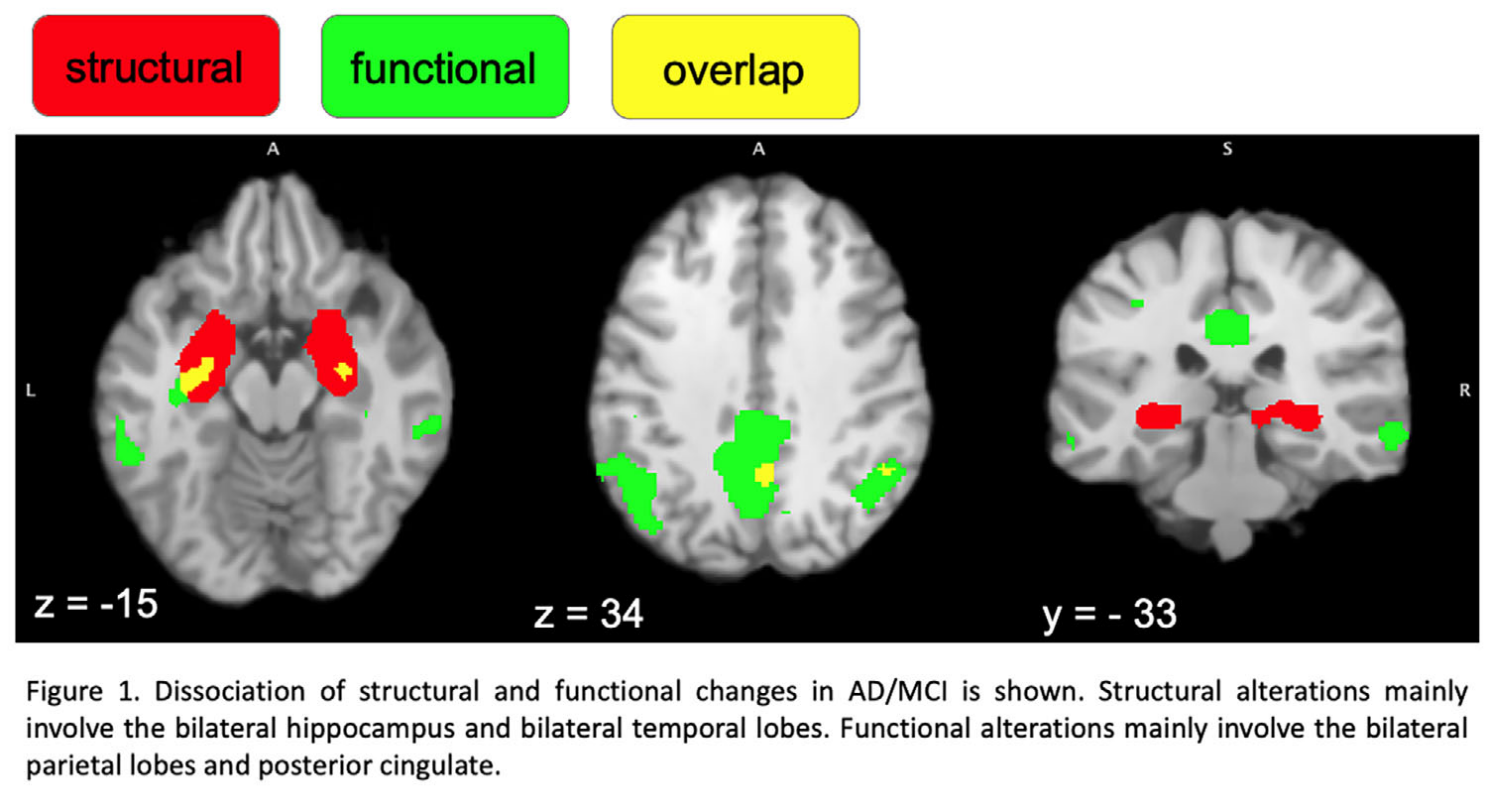# Dissociation of Structural and Functional Changes in Alzheimer’s Disease and Mild Cognitive Impairment

**DOI:** 10.1002/alz.093740

**Published:** 2025-01-09

**Authors:** Annie Dang, Di Wang, Jonathan Towne, Mohamad Habes, Peter T Fox

**Affiliations:** ^1^ UT Health San Antonio, San Antonio, TX USA; ^2^ Glenn Biggs Institute for Alzheimer’s & Neurodegenerative Diseases, University of Texas Health Sciences Center at San Antonio, San Antonio, TX USA

## Abstract

**Background:**

The Amyloid‐Tau‐Neurodegeneration (ATN) biomarker framework for Alzheimer’s disease (AD) indicates binary (presence/absence) designations for each type of pathology, without regard for anatomical distribution. Neurodegeneration is designated as positive if atrophy or hypometabolism are found on imaging. However, Clifford Jack et al., 2016 noted that atrophy and hypometabolism were differently distributed and referenced each to different co‐localized pathologies. Thus, there exists a need to further characterize atrophy and hypometabolic changes in AD, with the goal of advancing the application of anatomically‐based biomarkers in the ATN framework.

**Method:**

Query of the BrainMap databases of published, group‐wise neuroimaging, case‐control contrasts was used to identify AD and mild cognitive impairment (MCI) studies for meta‐analysis. The voxel‐based morphometry (VBM) and voxel‐based physiology (VBP) databases were used to identify studies involving atrophy and hypometabolism respectively. 157 VBM contrasts (110 AD, 47 MCI) and 146 VBP contrasts (88 AD, 58 MCI) were identified. Activation likelihood estimation coordinate‐based meta‐analysis was performed separately for VBM and VBP, to identify cross‐study convergence of brain alteration patterns. Mango was then used to visualize results and quantify spatial overlap between VBM and VBP.

**Result:**

Structural (atrophy) and functional (hypophysiology) neurodegenerations in AD/MCI exhibit markedly different neuroanatomical distributions (Figure 1). Structural abnormalities chiefly involve the bilateral hippocampus and bilateral temporal lobes; functional abnormalities chiefly involve the bilateral parietal lobes and posterior cingulate. There is a small overlap (2184 mm3) between VBM and VBP, accounting for 10.1% of VBM and 7.1% of VBP.

**Conclusion:**

VBM and VBP patterns of alteration appear distinct, aligning with the anterior and posterior default mode network respectively. This dissociation may reflect distinct underlying neuropathologies. We suggest that this knowledge can be used to advance the application of anatomically‐based biomarkers in the ATN framework. Network modeling of VBM and VBP data is currently ongoing.